# An Enzyme That Oversees RNA Quality Control

**DOI:** 10.1371/journal.pbio.0030191

**Published:** 2005-04-19

The path from DNA to RNA to protein sounds straightforward enough, but few processes in the life of a cell could be called simple. Gene expression is a complex, highly regulated affair that involves the activity of discrete teams of molecular manipulators at key steps in the protein production pathway. As soon as transcription begins, RNA processing machinery sets to work on messenger RNA (mRNA) precursors in the nucleus, proofreading the RNA copy, stabilizing the elongating transcripts, and making sure mRNAs reach the translation machinery in the cytoplasm.

A key step in RNA processing involves the addition of a string of adenine nucleotides to the 3′ end of the growing transcript, a modification called polyadenylation. (Each RNA chain has what's called a 5′ end and a 3′ end, which relates to the chemical polarity of the nucleotides.) In eukaryotes like yeast and humans, polyadenylation helps to stabilize the mRNA transcript and to ensure its export from the nucleus. Once in the cytoplasm, the mRNAs' poly(A) tails interact with components of the translation apparatus and facilitate protein synthesis. Polyadenylation is mediated by a class of enzymes called poly(A) polymerases (PAPs), which typically act in concert with other proteins in the cell's nucleus.

Just three years ago, a new class of PAPs was discovered that belong to the Trf4/5 family of yeast proteins and are found in the cytoplasm, rather than the nucleus, of the cell. Unlike the nuclear PAPs, in which one protein contains both catalytic activity and an RNA-binding domain, the new class relies on two or more protein subunits to carry out these tasks. It's been suggested that these enzymes stabilize specific mRNAs in the cytoplasm by extending their poly(A) tails. It's long been known that adding poly(A) tails to RNAs in prokaryotic bacteria promotes the degradation of defective RNAs, but the existence of a similar mechanism in yeast has just recently come to light. Previous genetic experiments on live yeast cells by James Anderson and collaborators suggested that when the protein Trf4p polyadenylated a particular type of abnormal transfer RNA (tRNA)—the intermediary that translates the nucleotide code into the amino acid code—the tRNA was destroyed. In a new study, Walter Keller and colleagues investigate the biochemistry, composition, and function of Trf4p in the brewer's yeast Saccharomyces cerevisiae, and find evidence that Trf4p-mediated polyadenylation plays a role in RNA quality control in eukaryotes.[Fig pbio-0030191-g001]

**Figure pbio-0030191-g001:**
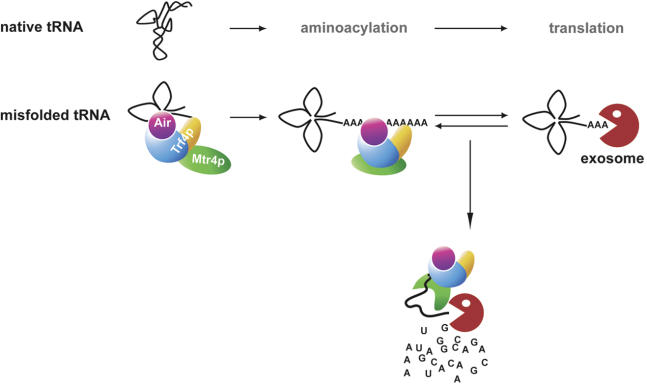
Model of the tRNA surveillance mechanism via polyadenylation of misfolded RNA by Trf4 complex leading to degradation by the exosome

After showing that mutating two amino acid residues in the predicted catalytic center of Trf4p eliminates its activity, Keller and coworkers determined that the enzyme forms a PAP complex with three other proteins (including two putative RNA-binding proteins, Air1p and Air2p, and a putative RNA-unwinding enzyme called Mtr4p that most likely functions to unwind structured regions in the RNAs). They also showed that the Trf4p complex selectively targets and successfully polyadenylated only tRNA molecules that were either lacking the chemical modifications required for normal folding or that were misfolded by mutation. Polyadenylation appears to tag the aberrant RNA as damaged goods, signaling the cell's nuclear molecule-degradation complex, the exosome, to initiate destruction.

Keller and colleagues propose that the Trf4p complex recognizes structural defects in RNA, prompting the Trf4p subunit to add poly(A) tails to the RNA, which initiates RNA degradation. In this model, Trf4p, along with either Air1p or Air2p, interacts with the RNA enzyme Mtr4p, which physically connects the tRNA-Trf4-PAP complex to the exosome. These results suggest that Trf4-PAP monitors the quality of tRNAs by detecting misfolded RNAs and engineering their destruction before they can gum up the works of protein assembly.

That the polyadenylation pathway for discarding defective tRNA appears in both bacteria and yeast suggests that this quality-control mechanism represents the ancient role for polyadenylation, the authors propose; the stabilization function of adding poly(A) tails may have arisen as eukaryotes evolved a nucleus and other organelles. Whether this notion or the model described here proves correct remains to be seen, and the authors outline a number of avenues for further study. Determining the structure of RNA–protein complexes, for example, and their binding properties and interactions, the authors argue, should elucidate the mechanism by which this RNA surveillance complex operates and what features of its RNA substrates it recognizes.

